# Blood pressure outcomes at 12 months in primary care patients prescribed remote physiological monitoring for hypertension: a prospective cohort study

**DOI:** 10.1038/s41371-023-00850-w

**Published:** 2023-07-21

**Authors:** Lucia C. Petito, Lauren Anthony, Yaw Peprah, Ji Young Lee, Jim Li, Hironori Sato, Stephen D. Persell

**Affiliations:** 1 Division of Biostatistics, Department of Preventive Medicine, Feinberg School of Medicine, Northwestern University, Chicago, IL USA; 2 Northwestern Medical Group Quality and Patient Safety, Northwestern Memorial Healthcare, Chicago, IL USA; 3 Division of General Internal Medicine, Department of Medicine, Feinberg School of Medicine, Northwestern University, Chicago, IL USA; 4 Strategic Clinical RD Department, Technology Development HQ, Omron Healthcare, Co., Ltd., Kyoto, Japan; 5 Product Innovation Department, Technology Development HQ, Omron Healthcare, Co., Ltd., Kyoto, Japan; 6 Center for Primary Care Innovation, Institute for Public Health and Medicine, Feinberg School of Medicine, Northwestern University, Chicago, IL USA

**Keywords:** Disease prevention, Medical research

## Abstract

Remote patient monitoring (RPM) for hypertension enables automatic transmission of blood pressure (BP) and pulse into the electronic health record (EHR), but its effectiveness in primary care is unknown. This pragmatic matched cohort study using EHR data compared BP outcomes between individuals prescribed RPM and temporally-matched controls from six primary care practices. We retrospectively created a cohort of 288 Medicare-enrolled patients prescribed BP RPM (cases) and 1152 propensity score-matched controls (1:4). Matching was based on age, sex, systolic blood pressure (SBP), marital status, and other characteristics. Outcomes at 3, 6, 9 and 12 months were: controlling high BP (most recent BP < 140/90 mm Hg), antihypertensive medication intensification, and most recent SBP assessed using: all measurements, and office measurements only. At baseline, RPM-prescribed patients and controls had similar ages and systolic BP. BP control diverged at 3 months (RPM: 72.2%, control: 51%, *p* < 0.001). This difference persisted but decreased over follow-up. After 12 months, the RPM-prescribed cohort had greater BP control (RPM: 71.5%, control: 58.1%, *p* < 0.001) and lower SBP (132.3 versus 136.5 mm Hg, *p* = 0.003) using all measurements, but they did not differ using only office measurements (12 month BP control: 60.8% versus 58.1%, *p* = 0.44; SBP: 135.9 versus 136.5 mm Hg, *p* = 0.91). At 12 months, the most recent BP measurements were more current for RPM-prescribed patients (median [IQR] 8 [0–109] versus 134 [56–239] days). Net increases in antihypertensive medications by 12 months were similar. Implementation of RPM in primary care could inform hypertension management strategies and increase hypertension control. Registration: ClinicalTrials.gov identifier: NCT05562921.

## Introduction

Hypertension control in the United States has recently declined [1]. Under experimental conditions, self-measurement of out-of-office blood pressure (BP) combined with tele-monitoring interventions has been shown to improve BP control [2–4]. Out-of-office measurement has been endorsed in multiple guidelines as a means to improve hypertension diagnosis and management [5–7].

In 2019, Medicare began covering Remote Physiological Monitoring or Remote Patient Monitoring (RPM) services that can be used to support the management of BP [8]. Previously, we conducted two pilot studies at six primary care practices where RPM was made available to clinicians [9, 10]. In those studies we generated pre-defined cohorts of hypertensive patients with Medicare insurance and propensity matched cohorts from other practices to demonstrate the effects of RPM availability within specific populations—a quasi-experimental intention-to-treat analysis designed to study the effect of making RPM available to clinicians. Those studies were designed to demonstrate population-level preliminary efficacy of making RPM available in primary care practices, and did show some population-level effects. But, because uptake occurred in small proportions of eligible patient populations only, it is difficult to ascribe these effects to RPM availability. To understand the impact of the prescription of a RPM device on patients, here we used similar methods to compare patients who were prescribed RPM at the six pilot clinics to propensity score-matched cohorts of controls from those same clinics.

We conducted a pragmatic matched cohort study in hypertensive Medicare patients at six primary care practices that participated in two pilot studies that have been previously described [9, 10]. Two practices implemented RPM with support of nurse care coordination and four implemented it exclusively at the discretion of the primary care clinicians. We assembled an intervention cohort consisting of all patients prescribed the blood pressure RPM system, and created a temporally-matched cohort of patients who did not receive a RPM prescription. We compared BP-related outcomes, including hypertension control, SBP achieved, and antihypertensive medication intensification, over 12 months in these cohorts.

## Patients and methods

This study was conducted at primary care practices within Northwestern Medicine, a health system in the Chicago Illinois region. Six practices (three in Chicago, three suburban) were approached based on convenience. Patients were prescribed RPM between November 18, 2020 and August 14, 2021; these patients and their matched controls were followed for 12 months after the RPM prescription. The system uses the Epic (Epic Systems Corp., Verona, WI) electronic health record (EHR), from which all study data, including data used for identifying the comparison cohorts and outcome data, were abstracted.

This study was approved by the Northwestern University Institutional Review Board. All data used were obtained in the course of routine medical care, extracted from EHR data copied to a data warehouse, and used with a waiver of informed consent. Reporting follows the STROBE recommendations [11].

### Study participants

We used propensity scores to match patients who received a prescription for RPM to control patients at the same clinics. The cohorts were held fixed for the study duration. At the start date for each RPM-prescribed patient or matched controls, all patients were required to be aged 65–85 years, have Medicare or Medicare Advantage insurance, and have had at least one office or telehealth visit in the preceding year. Outcome ascertainment occurred in the 12 month period after the index date for all patients.

#### Creation of the matched cohort

We sought to generate a matched cohort that was similar in terms of blood pressure level, clinical risk, renal function, demographics, and recent primary care utilization. Matched controls were selected based on demographics (sex, age, race/ethnicity), presence of comorbidities (diabetes mellitus, atherosclerotic cardiovascular disease), laboratory values (total cholesterol, HDL cholesterol, creatinine), healthcare use (visits in prior year) and BP (recent in-office SBP, antihypertensive medication use). To ensure controls were temporally matched to RPM recipients, time of most recent office visit for controls was matched to the time of RPM prescription (month of index visit). Taking advantage of the longitudinal nature of the EHR data, controls could contribute as a match for more than one intervention patient—they became newly eligible at each primary care visit. Supplementary Table S1 describes the matching characteristics. A greedy algorithm was used to select four controls for each RPM-prescribed patient based on propensity scores estimated from a main-terms logistic regression model [12, 13]. Supplementary Fig. 1 visualizes covariate balance between the cohorts before and after matching.

### Study measurements

#### Intervention: remote patient monitoring

We integrated Omron VitalSight^TM^ (Omron Healthcare, Inc., Hoffman Estates, IL) remote patient monitoring into the Northwestern Medicine Epic EHR (Epic Systems Co. Verona, WI) to automatically transmit BP and pulse data to patient records [9]. Primary care physicians at the two clinics participating in the initial pilot study had the option to prescribe RPM starting November 18, 2020. Decision support (a passive “Best Practice Advisory” in Epic) notified physicians when patients met study criteria for RPM (diagnosed hypertension, Medicare insurance). Omron Healthcare was notified of orders electronically and sent patients a Bluetooth^TM^-enabled automated BP monitor, cuff, cellular data transmitter and optional scale if selected by the ordering clinician. Two clinics in the second pilot had similar processes implemented starting February 15, 2021; two additional clinics also received care coordination support from nurses, which is more fully described elsewhere [10].

#### Outcomes

The primary effectiveness outcome, measured at 3, 6, 9 and 12 months after baseline, was the Controlling High Blood Pressure performance measure—National Quality Forum Measure 0018 (NQF0018)—most recent BP < 140/90 mm Hg within the prior 12 months [14, 15]. Patients without a BP measurement in the preceding 12 months did not meet the measure. The lowest systolic and lowest diastolic BPs from the day with the most recent BP measurement and was used and included office and RPM-obtained BPs.

Secondary outcomes included most recent primary care in-office SBP and antihypertensive medication intensification. If an individual had multiple in-office measurements from the most recent BP measurement day, we used their average. Patients with no BP measured in the year preceding the measurement date were excluded due to missingness. The absolute number of antihypertensive medication intensifications were determined from the EHR medication list. Net increases in antihypertensive medications were considered present if the number of antihypertensive drug classes added or with a dose increased minus the number of classes discontinued or with a dose decreased was greater than zero.

To evaluate the influence that remote readings had on controlling high blood pressure and systolic blood pressure, we compared versions of these metrics that used office and RPM values with those that used primary care office BPs exclusively. We also examined how recently the blood pressures had been obtained at 12 months in the office-only and RPM-derived measures.

#### Additional study variables

We abstracted demographic characteristics, diagnoses, and RPM and office vital signs from the EHR (Supplementary Table 1). Among patients prescribed RPM, we measured whether they used the device, time to uptake (days between prescription and first reading), intensity of use (mean readings per 30 days), and duration of use (time from first reading to a reading with no subsequent reading for 30 days). To examine healthcare utilization, we measured primary care office visits, patient portal messages, and telephone encounters during the study period.

### Statistical analysis

We constructed the 1:4 propensity score matched cohort as described above. We used means and proportions to describe demographic characteristics, and healthcare usage of patients enrolled in RPM and the control cohort.

At 3, 6, 9 and 12 months following the index date (prescription date for RPM or date of primary care visit for matched controls), we calculated averages and proportions as appropriate for continuous and binary outcomes. Generalized linear models were used to estimate differences between RPM-prescribed patients and matched controls in means of continuous variables (identity link) and differences in log-odds for categorical variables (logit link); a random effect for patient was included to account for correlation between observations on the same control who was matched to multiple RPM-prescribed patients. For utilization comparisons we used chi square or Fisher exact tests.

Two of the six pilot practices that used RPM employed a care coordination co-intervention, so we performed an exploratory subgroup analysis examining blood pressure outcomes stratified by whether or not patients were in care coordination practices.

After observing large differences between BP outcomes obtained with and without the use of RPM values, we performed an exploratory analysis. At baseline, 3, 6, 9 and 12 months we examined RPM-prescribed patients who had: at least one in-office SBP measured up to 30 days before or after the time point (indexed to the individual-specific date of initial RPM prescription). We then evaluated remote SBP measurements taken up to 14 days before or after the patient’s corresponding office visit date where blood pressure was measured. The office SBP came from the visit closest to the outcome timepoint (within 30 days before or after); if multiple SBP measurements were available on that date, their mean was used. Remote SBP was calculated as the mean of all remote SBPs within 14 days before or after each patient’s corresponding office visit date. We used paired *t*-tests to compare remote and in-office systolic blood pressures within individuals.

Analyses used SAS 9.4. Significance was assessed using a 5% type-I error rate (conservatively Bonferroni corrected to 1.25% for the 4 primary outcomes); both *p* values and 95% confidence intervals were used in interpretation.

## Results

### Patient characteristics

Characteristics of the 288 RPM-prescribed patients and 1152 matched control patients (1089 unique patients—63 were matched to multiple RPM-prescribed patients beginning at different primary care visits) were similar (Table 1). Mean (SD) age was 73.6 (7.4) years in the RPM group and 73.8 (7.9) in controls. Approximately two-thirds were female, and 81.6% in the RPM and 84.0% in the controls had an active prescription for antihypertensive medication at their index visit. Mean baseline blood pressures were similar: systolic/diastolic BP of 143/77 mm Hg in RPM-prescribed patients and 141/76 mm Hg in controls.

**Table 1:** Baseline characteristics of remote physiological monitoring (RPM)-prescribed patients and matched controls.

Characteristic	RPM prescribed (*N* = 288)	Matched controls^a^ (*N* = 1152)
Demographics^b^		
Age, years, mean (SD)	73.6 (7.4)	73.8 (7.9)
Female, *n* (%)	195 (67.7)	814 (70.7)
Race/ethnicity, *n* (%)		
Non-Hispanic White	204 (70.8)	797 (69.2)
Other	84 (29.2)	355 (30.8)
Marital status, *n* (%)		
Married/Partnered	135 (46.9)	569 (49.4)
Single/Separate/Widowed	153 (53.1)	583 (50.6)
Clinical characteristics^b^		
Recent BP		
Available at index visit, *n* (%)	231 (80.2)	924 (80.2)
SBP, mean (SD)	142.7 (19.5)	141.2 (18.7)
DBP, mean (SD)	77.3 (9.8)	75.9 (9.5)
Historical BP, *n*		
Available in 2 years prior to index visit, *n* (%)	273 (94.8)	1092 (94.8)
SBP, mean (SD)	139.9 (18.9)	140.2 (19.5)
DBP, mean (SD)	76.1 (7.4)	75.7 (8.1)
Antihypertensive medication prescribed, *n* (%)	235 (81.6)	968 (84.0)
Diabetes mellitus, *n* (%)	62 (21.5)	230 (20.0)
ASCVD, *n* (%)	33 (11.5)	126 (10.9)
Laboratory values^c^		
Total cholesterol, mean (SD)	173.6 (40.3)	172.4 (41.1)
HDL cholesterol, mean (SD)	57.5 (17.1)	57.5 (16.6)
eGFR, mean (SD)	71.0 (17.9)	70.6 (19.4)
Healthcare utilization^d^		
No. of visits to primary care clinics, *n* (%)		
0	39 (13.5)	125 (10.9)
1	75 (26.0)	309 (26.8)
2	75 (26.0)	285 (24.7)
3	39 (13.5)	162 (14.1)
4 +	60 (20.8)	271 (23.5)

*RPM* remote physiologic monitoring, *SBP* systolic blood pressure, *DBP* diastolic blood pressure, *ASCVD* atherosclerotic cardiovascular disease, *HDL* high density lipoprotein.

^a^The 1152 matched controls represent 1089 unique patients, as controls were eligible to be matched at each primary care visit they had during the study period. Standardized mean differences are reported in Fig. S1.

^b^These characteristics were measured as of 11/19/2020.

^c^Total cholesterol, HDL cholesterol, and eGFR were measured in 85%, 86%, and 93% of RPM users and 87%, 87%, and 94% of matched controls, respectively.

^d^Visits to primary care clinics in the 1 year prior to RPM becoming available at NM: 11/19/2019–11/19/2020.

### RPM usage

Table 2 presents characteristics of RPM use in the first year after prescription among the 288 patients prescribed RPM. Two hundred and forty patients (83%) transmitted at least one blood pressure, 233 (81%) transmitted at least 12, and the median time to last RPM use was 361 days (inner quartile range [IQR]: 235–365 days). Most RPM users transmitted a BP reading at least every other day in months when they used the device (median 26 readings, IQR: 14–40 readings).

**Table 2:** Utilization of remote physiological monitoring (RPM) device in first 12 months after prescription among RPM-prescribed patients.

	RPM prescribed (*n* = 288)
Patients with any remote BP transmitted, *n* (%)	240 (83.3)
Patients with at least 12 remote BP transmitted, *n* (%)	233 (80.9)
Time to initial use (days), median (IQR)	8 (6, 16)
Time to last use (days), median (IQR)	360.5 (235, 365)
Average no. of RPM transmissions per month in months when used, median (IQR)	26 (14, 40)
Total no. of months with at least one transmission during study period, median (IQR)	11 (6, 12)

*RPM* remote physiologic monitoring, *IQR* inner quartile range, *no.* number.

### Blood pressure outcomes within 12 months of index date

The prevalence of Controlling High Blood Pressure was similar in both cohorts at baseline (35% in RPM-prescribed versus 39% in controls, *p* = 0.24, Table 3). After RPM prescription, the prevalence of Controlling High Blood Pressure doubled in the RPM-prescribed group (35% to 70–73%). Controls also saw an increase in BP control (39% to 51–59%). Compared with controls, a greater proportion of RPM-prescribed patients had controlled high blood pressure at 3, 6, 9 and 12 months (month-specific ORs ranged 1.7–2.5; *p* < 0.001). RPM-prescribed patients and controls had similar systolic BP at the index visit (142.7 mm Hg versus 141.2 mm Hg, *p* = 0.16, Table 3). After 12 months of device use, a 10.4 mm Hg decrease in systolic BP was observed in the RPM-prescribed patients, compared with a 4.7 mm Hg drop among controls. On average, RPM-prescribed patients had lower systolic BP than matched controls after 12 months (marginal mean difference [MMD] −3.8 mm Hg; 95% CI: −6.2, −1.3; *p* = 0.003).

**Table 3:** Blood pressure (BP) measurements assessed over 12 months in patients prescribed remote patient monitoring (RPM) compared with matched controls.

	Controlling high blood pressure, %^a^				Systolic blood pressure, mm Hg^b,c^			
	RPM prescribed *N* = 288	Matched controls *N* = 1152	Odds ratio (95% CI)	*p*^d^	RPM prescribed *N* = 288	Matched controls *N* = 1152	Difference (95% CI)	*p*^d^
Baseline: Blood pressure metrics (in-office)								
Index	35.4	39.2	0.8 (0.6, 1.1)	0.24	142.7 (19.5)	141.2 (18.7)	2.0 (−8.8, 9.8)	0.16
Outcomes: Blood pressure metrics over time (in-office and RPM)^e^								
3 months	72.2	51.0	2.5 (1.9, 3.4)	**<0.001**	134.2 (17.9)	140.0 (18.9)	−5.4 (−7.9, −2.9)	<0.001
6 months	72.9	56.6	2.1 (1.5, 2.8)	**<0.001**	132.6 (18.0)	137.5 (18.6)	−4.4 (−6.9, −1.9)	<0.001
9 months	70.1	58.5	1.7 (1.2, 2.2)	**0.001**	133.2 (18.5)	136.3 (18.0)	−2.6 (−5.0, −0.2)	0.04
12 months^d^	71.5	58.1	1.8 (1.3, 2.4)	**<0.001**	132.3 (17.9)	136.5 (18.4)	−3.8 (−6.2, −1.3)	0.003
Outcomes: Blood pressure metrics over time (in-office)								
3 months	50.0	51.0	1.0 (0.7, 1.3)	0.75	140.8 (19.1)	140.0 (18.9)	1.2 (−1.3, 3.8)	0.33
6 months	59.0	56.6	1.1 (0.8, 1.5)	0.50	137.7 (17.7)	137.5 (18.6)	0.6 (−1.8, 3.1)	0.62
9 months	58.3	58.5	1.0 (0.8, 1.3)	0.92	137.2 (18.0)	136.3 (18.0)	1.4 (−1.0, 3.8)	0.25
12 months	60.8	58.1	1.1 (0.8, 1.5)	0.44	135.9 (18.5)	136.5 (18.4)	−0.1 (−2.6, 2.3)	0.91

*RPM* remote physiologic monitoring, *CI* confidence interval.

^a^The lowest recorded SBP and DBP were used if there were multiple measurements on the same date.

^b^The average daily BP was used if there were multiple measurements on the same date.

^c^This sample underwent minor attrition between baseline and follow-up: 11 (3.8%), 8 (2.8%), 8 (2.8%), and 10 (3.5%) of RPM-prescribed patients and 84 (7.3%), 83 (7.2%), 91 (7.9%), and 85 (7.4%) of matched controls for the systolic blood pressure outcomes assessed at 3, 6, 9, and 12 months, respectively. Patients without a blood pressure in the preceding 12 months we counted as not meeting the Controlling High Blood Pressure measure.

^d^*p* values for differences in means or proportions were calculated from generalized linear mixed models, with a random intercept for patient.

^e^Bolded *p* values indicate statistical significance at a 5% type-I error rate after Bonferroni correction of the primary outcome.

In a sensitivity analysis where we only included in-office BP measurements, the cohorts had similar prevalences of Controlling High Blood Pressure and systolic BP measurements at each outcome assessment (Table 3).

### Antihypertensive medication changes and healthcare utilization

We compared medication changes and the proportion of patients with a net increase in prescribed antihypertensive medications between RPM-prescribed patients and controls. The distributions of antihypertensive medication changes and in the proportion with a net antihypertensive medication increase was similar in both cohorts (Table 4). After 1 year, 37.5% of the RPM and 41.1% of the control cohort had a net increase in antihypertensive medication therapy (*p* = 0.33).

**Table 4:** Medication changes and healthcare utilization during the 12-month study period.

	RPM prescribed (*N* = 288)	Matched controls (*N* = 1152)	*p* value
Antihypertensive medication changes			
Net antihypertensive medication intensification, *n* (%)^a^			0.35
−5	–	2 (0.2)	
−4	–	3 (0.3)	
−3	3 (1.0)	10 (0.9)	
−2	8 (2.8)	24 (2.1)	
−1	34 (11.8)	118 (10.2)	
0	135 (46.9)	522 (45.3)	
1	76 (26.4)	363 (31.5)	
2	27 (9.4)	81 (7.0)	
3	2 (0.7)	15 (1.3)	
4	3 (1.4)	8 (0.7)	
5	–	2 (0.2)	
7	–	2 (0.2)	
Net antihypertensive medication increase, *n* (%)^b^	108 (37.5)	473 (41.1)	0.33
Healthcare utilization			
In-person/telehealth encounters, *n* (%)			<0.001
0	2 (0.7)	72 (6.3)	
1	41 (14.2)	228 (19.8)	
2	71 (24.7)	270 (23.4)	
3	57 (19.8)	217 (18.8)	
4+	117 (40.6)	365 (31.7)	
Patient portal encounters, *n* (%)			<0.001
0	45 (15.6)	340 (29.5)	
1	25 (8.7)	139 (12.1)	
2	19 (6.6)	135 (11.7)	
3	27 (9.4)	91 (7.9)	
4+	172 (59.7)	447 (38.8)	
Telephone encounters, *n* (%)			0.06
0	53 (18.4)	301 (26.1)	
1	49 (17.0)	190 (16.5)	
2	40 (13.9)	164 (14.2)	
3	32 (11.1)	123 (10.7)	
4+	114 (39.6)	374 (32.5)	

*RPM* remote physiologic monitoring.

^a^Number of antihypertensive medication classes added or increased minus the number discontinued or decreased.

^b^Net antihypertensive medication intensification greater than zero.

To better understand the implication of RPM use on health system resources, we compared the distribution of in-person/telehealth visits, patient portal use and telephone encounters. RPM-prescribed patients had more in-person/telehealth, patient portal, and telephone encounters than controls (*p* < 0.001, *p* < 0.001, and *p* = 0.06, respectively) (Table 4).

### Exploratory analysis: effect of care coordination on blood pressure outcomes

Access to care coordination had a positive impact on Controlling High Blood Pressure. The prevalence of control was much lower in RPM-prescribed patients at care coordination clinics at baseline than non-care coordination clinics (24% versus 41%, Table S2). After RPM prescription, the prevalence of Controlling High Blood Pressure tripled at care coordination clinics (24% to 68–83%), whereas it only increased by about 75% at non-care coordination clinics (41% to 67–71%). After 12 months, compared with controls at care coordination clinics, RPM-prescribed patients had three times the odds of Controlling High Blood Pressure (95% CI 1.6, 5.5, *p* = 0.004); at clinics without care coordination, the odds of controlling high blood pressure was 1.4 times greater in RPM-prescribed patients (95% CI 1.0, 2.1, *p* = 0.06) (*p* = 0.03 for treatment effect heterogeneity).

RPM-prescribed patients at care coordination and non-care coordination clinics had similar systolic BP at index visit (mean 143 mm Hg, Table S3). Similar decreases in systolic BPs were observed in RPM-prescribed patients with and without care coordination. After 12 months, we did not observe meaningful effect heterogeneity by clinic type (at care coordination clinics, MMD −4.3 mm Hg; 95% CI: −9.1, 0.5; *p* = 0.07; at non-care coordination clinics, MMD −4.0 mm Hg; 95% CI −7.1, −0.8; *p* = 0.02) (*p* = 0.89 for treatment effect heterogeneity).

### Exploratory comparisons of remote and in-office systolic blood pressure

Comparisons of in-office systolic blood pressures with remote SBP for individuals who had both types of readings within the examined time intervals showed that SBP decreased over time for both measurement types and that there were no significant differences between the two (Table S4).

For the 12-month outcomes, the most recent blood pressures measurements were much more current for RPM-prescribed patients—median 8 days, IQR (0–109) versus 134 days (56–239) for controls. The time since most recent primary care office BP was similar (Table S5).

## Discussion

To our knowledge, this is the largest follow-up study of an RPM intervention in hypertensive patients in routine primary care. Compared with controls, RPM-prescribed patients were more likely to meet the Controlling High Blood Pressure metric after 12 months. We leveraged EHR data to examine blood pressure outcomes in a cohort of 288 Medicare patients prescribed RPM and compared them to 1152 temporally-matched control patients from the same practices, minimizing bias introduced by differences in quality of care accessed and provider variation.

These findings suggest that the incorporation of RPM into the care of hypertensive patients has the potential to quickly improve hypertension control. We observed a marked decrease in systolic BP within the first 3 months of RPM use (8 mm Hg compared with 1 mm Hg in matched controls, Table 3). This drop continued over 12-months to more than 10 mm Hg in RPM-prescribed patients. We hypothesize that improvement with RPM could be due to changes in behaviorals such as diet, exercise or medication adherence. Alternatively, differences could be due to differences in antihypertensive medication prescribing. As we did not observe meaningful differences in medication adjustments, this substantial improvement in Controlling High Blood Pressure could be from differences in the unmeasured behavioral factors related to blood pressure, or possibly from more appropriate medication changes (both intensification in patients with truly elevated blood pressure and de-intensification in patients with low blood pressure) among RPM patients who had more frequent, and potentially more reliable, blood pressure assessments.

We explored other factors which could account for observed differences. We speculated that there could be unmeasured white-coat hypertension or white-coat effect among controls, and that their true blood pressures were similar to those of the RPM-prescribed patients. However, the similarities in systolic blood pressure for patients who had in- and out-of-office measurements at the same times refutes this idea. An important difference between groups was that blood pressure readings were much more current in the RPM group. Another potential explanation for between-group differences that we cannot directly assess is that patients in the control cohort who did not return for recent office readings experienced an unmeasured downward trend in blood pressure that went uncaptured because home readings were not recorded.

The Controlling High Blood Pressure measure is used in multiple quality reporting programs [14, 15] and U.S. performance has stagnated [1, 15]. Our findings suggest that uptake of RPM in hypertensive patients can have important short and intermediate-term effects on this measure. In addition, because of increased accuracy from a greater number of measurements and the ability to obtain more current measurements between visits, we speculate that an increase in remote monitoring could allow for population health management programs to more accurately target poorly controlled patients.

These findings should be viewed in the context of the existing literature on hypertension management using RPM. Prior studies showed interventions that only promoted self monitoring had little effect [16]. More intensive strategies that incorporated remote monitoring and specific care teams to provide telehealth counseling and medication adjustments have been some of the most powerful interventions to date [4, 16–19]. Here the subgroup who received counseling and support from nurses had larger blood pressure improvements which is consistent with prior studies. However, the care coordination team may have identified patients who would benefit most from RPM use, as indicated by the low prevalence of blood pressure control at the index visit (24% versus 41%, Table S2). Even without care coordination, RPM-prescribed patients had an 8 to 13% higher rate of BP control compared to controls, indicating that important differences were observed even without the care coordination co-intervention.

### Strengths and limitations

A major study strength was use of EHR data enabling a prospective cohort design and continuous follow-up for 12 months. Limitations included the infeasibility of randomization in this setting; since we conducted an observational study, patients selected for RPM may have differed systematically from others based on unmeasured characteristics. In particular, patients who were more likely to engage with their hypertension care may have been more likely to accept an offer for RPM from their physician. However, the matched design minimized the differences between patients on the multiple characteristics included in the matching algorithm. Second, patients who were not prescribed RPM may have been performing home monitoring that was not available in structured data in the EHR, preventing its incorporation in our analysis. Third, we do not know how generalizable these findings are to other patient populations; our patient population was 70% non-Hispanic White, and more diverse populations might respond differently. Fourth, although uptake of RPM once prescribed was high (>80%), we did not survey patients to investigate barriers or facilitators to initial RPM use.

## Conclusion

In this matched cohort study conducted with EHR data from six clinics, we observed higher prevalences of Controlling High Blood Pressure and lower systolic BP after 12 months among patients prescribed RPM compared with controls. RPM uptake was quite good: 80% of individuals prescribed an RPM device used it at least 12 times, and more than half were still using it 1 year later. Most recent blood pressures were much more current among RPM users. However, mechanisms by which RPM use improves BP are not clear. Implementation of RPM for BP monitoring should be considered for hypertensive patients as a result of these encouraging findings.

## Summary

### What is known about this topic

- Experimental hypertension management strategies using home blood pressure monitoring and specific care teams have successfully improved blood pressure.
- The effects of implementing hypertension remote monitoring into routine primary care are not known.

### What this study adds

- A large majority of patients prescribed remote monitoring became active users.
- Compared with propensity score-matched controls, primary care patients who were prescribed remote physiological monitoring for blood pressure were more likely to have controlled high blood pressure at 3 months and this difference persisted at 12 months.

### Supplementary information

Supplemental Material

## Data Availability

A deidentified dataset will be shared with other investigators on reasonable request to the corresponding author.

## References

[1] Muntner P, Hardy ST, Fine LJ, Jaeger BC, Wozniak G, Levitan EB (2020). Trends in blood pressure control among US adults with hypertension, 1999-2000 to 2017-2018. JAMA.

[2] Omboni S, Gazzola T, Carabelli G, Parati G (2013). Clinical usefulness and cost effectiveness of home blood pressure telemonitoring: meta-analysis of randomized controlled studies. J Hypertens.

[3] Duan Y, Xie Z, Dong F, Wu Z, Lin Z, Sun N (2017). Effectiveness of home blood pressure telemonitoring: a systematic review and meta-analysis of randomised controlled studies. J Hum Hypertens.

[4] Tucker KL, Sheppard JP, Stevens R, Bosworth HB, Bove A, Bray EP (2017). Self-monitoring of blood pressure in hypertension: a systematic review and individual patient data meta-analysis. PLoS Med.

[5] Shimbo D, Artinian NT, Basile JN, Krakoff LR, Margolis KL, Rakotz MK (2020). Self-measured blood pressure monitoring at home: a joint policy statement from the American Heart Association and American Medical Association. Circulation.

[6] Whelton PK, Carey RM, Aronow WS, Casey DE, Collins KJ, Dennison Himmelfarb C (2017). ACC/AHA/AAPA/ABC/ACPM/AGS/APhA/ASH/ASPC/NMA/PCNA guideline for the prevention, detection, evaluation, and management of high blood pressure in adults: executive summary: a report of the American College of Cardiology/American Heart Association Task Force on clinical practice guidelines. Circulation.

[7] Williams B, Mancia G, Spiering W, Agabiti Rosei E, Azizi M, Burnier M (2018). Practice guidelines for the management of arterial hypertension of the European Society of Hypertension and the European Society of Cardiology: ESH/ESC Task Force for the management of arterial hypertension. J Hypertens.

[8] Centers for Medicare and Medicaid Services. Rules and Regulations 42 CFR Parts 403, 409, 410, 411, 414, 415, 416, 418, 424, 425, 489, and 498 [CMS–1715–F and IFC]. Federal Register. 2019;84:62568-63563.

[9] Petito LC, Anthony L, Peprah Y, Lee JY, Li J, Sato H (2023). Remote physiologic monitoring for hypertension in primary care: a prospective pragmatic pilot study in electronic health records using propensity score matching. JAMIA Open.

[10] Persell SD, Petito LC, Anthony L, Peprah Y, Lee JY, Campanella T (2023). Prospective cohort study of remote patient monitoring with and without care coordination for hypertension in primary care. Appl Clin Inform.

[11] von Elm E, Altman DG, Egger M, Pocock SJ, Gøtzsche PC, Vandenbroucke JP (2014). STROBE Initiative. The strengthening the reporting of observational studies in epidemiology (STROBE) statement: guidelines for reporting observational studies. Int J Surg.

[12] Rosenbaum PR, Rubin DB (1985). Constructing a control group using multivariate matched sampling methods that incorporate the propensity score. Am Stat.

[13] Austin PC (2014). A comparison of 12 algorithms for matching on the propensity score. Stat Med.

[14] Centers for Medicare and Medicaid Services. Quality Payment Program. 2019. https://qpp.cms.gov/docs/QPP_quality_measure_specifications/CQM-Measures/2020_Measure_236_MIPSCQM.pdf.

[15] National Committee for Quality Assurance. Controlling High Blood Pressure. 2023. www.ncqa.org/hedis/measures/controlling-high-blood-pressure/.

[16] Margolis KL, Asche SE, Bergdall AR, Dehmer SP, Groen SE, Kadrmas HM (2013). Effect of home blood pressure telemonitoring and pharmacist management on blood pressure control: a cluster randomized clinical trial. JAMA.

[17] Margolis KL, Asche SE, Dehmer SP, Bergdall AR, Green BB, Sperl-Hillen JM (2018). Long-term outcomes of the effects of home blood pressure telemonitoring and pharmacist management on blood pressure among adults with uncontrolled hypertension: follow-up of a cluster randomized clinical trial. JAMA Netw Open.

[18] Green BB, Cook AJ, Ralston JD, Fishman PA, Catz SL, Carlson J (2008). Effectiveness of home blood pressure monitoring, web communication, and pharmacist care on hypertension control: a randomized controlled trial. JAMA.

[19] Green BB, Anderson ML, Ralston JD, Catz SL, Cook AJ (2013). Blood pressure 1 year after completion of web-based pharmacist care. JAMA Intern Med.

