# Supplementary material for: Blood pressure outcomes at 12 months in primary care patients prescribed remote physiological monitoring for hypertension: a prospective cohort study
Source: J Hum Hypertens. 2023 Jul 21;37(12):1091–7. doi: 10.1038/s41371-023-00850-w (PMC10739223; doi:10.1038/s41371-023-00850-w)
Supplement: Supplementary file 1 — Supplemental Material [file 41371_2023_850_MOESM1_ESM.docx]

**Supplemental Information**

**Table S1. Description of variables included in propensity score**

| **Variable Description** | **Possible values** |
| --- | --- |
| *Demographics* |  |
| Female sex | Y/N, at birth |
| Age | Continuous, years |
| Race/ethnicity | NH White, NH Black, NH Asian, NH American Indian or Alaska Native, NH Other |
| *Comorbid conditions* |  |
| Diabetes mellitus | Y/N, ever |
| Atherosclerotic cardiovascular disease (ASCVD) | Y/N, ever |
| *Laboratory values* |  |
| Total cholesterol | Measured? Y/N  If Y, value, mg/dL |
| HDL cholesterol | Measured? Y/N  If Y, value, mg/dL |
| Creatinine (to calculate eGFR) | Measured? Y/N |
| eGFR (calculated from creatinine) | Value, mg/dL/m3 |
| *Recent BP Information* |  |
| Was an in-office SBP measurement taken at the index visit (time of prescription of RPM for intervention; most recent office visit for control)? | Y/N  If Y, value, mm Hg |
| Was an in-office SBP measurement taken at a visit that occurred 6-24 months prior to the index visit (time of prescription of RPM for intervention; most recent office visit for control)? | Y/N  If Y, value, mm Hg |
| Active prescription for at least one antihypertensive medication at the time of the index visit | Y/N |
| *Visit Timing Information* |  |
| No. of visits at primary care clinics within one year prior to index visit | Categorical |
| Month of index visit | Categorical |

**Figure S1. Covariate balance before and after matching**


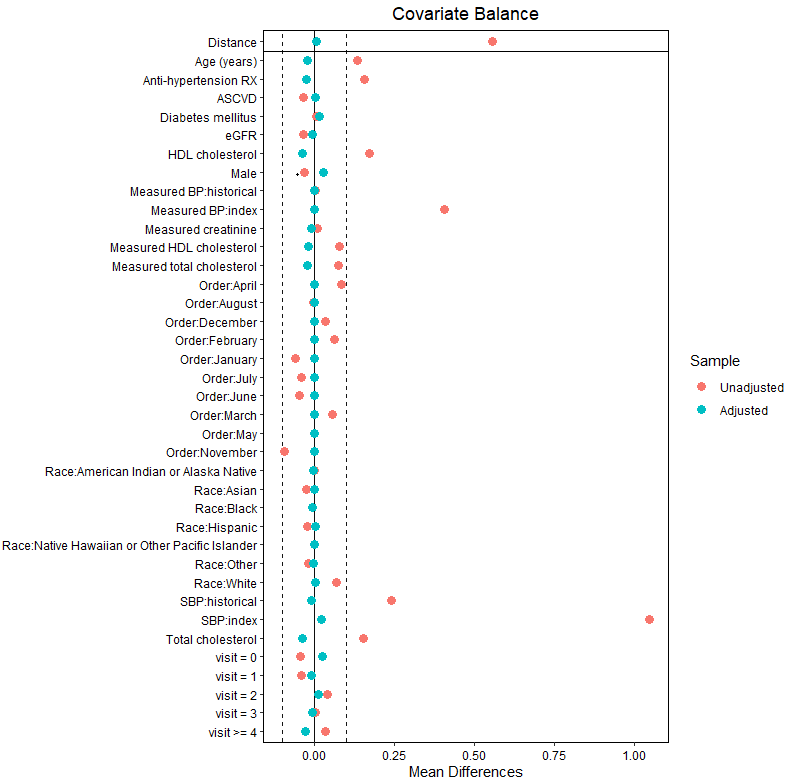
Dotted lines represent the region within 0.1 units for standardized mean differences (SMD) for each covariate. Red dots represent the SMD prior to matching, and blue dots represent the SMD after matching.

**Table S2.** **Controlling High Blood Pressure (BP) assessed over 12 Months in patients prescribed remote patient monitoring (RPM) compared with matched controls, stratified by whether clinic used care coordination.**

|  | **Controlling High Blood Pressure, %^a^**  **Care Coordination** | | | | **Controlling High Blood Pressure, %^a^**  **No Care Coordination** | | | |
| --- | --- | --- | --- | --- | --- | --- | --- | --- |
|  | RPM Prescribed N = 97 | Matched controls N = 388 | Odds Ratio (95% CI) | P^b^ | RPM Prescribed  N = 191 | Matched controls N = 764 | Odds Ratio  (95% CI) | P^b^ |
| ***Baseline: Blood Pressure Metrics (In-Office)*** | | | | |  | | | |
| Index | 23 (23.7) | 107 (27.6) | 0.8 (0.4, 1.5) | 0.47 | 79 (41.4) | 345 (45.2) | 0.9 (0.6, 1.2) | 0.36 |
| ***Outcomes: Blood Pressure Metrics Over Time (In-Office and RPM)*** | | | | |  | | | |
| 3 mo | 80 (82.5) | 176 (45.4) | 5.7 (2.9, 11.2) | <.001 | 128 (67.0) | 411 (53.8) | 1.7 (1.2, 2.5) | 0.004 |
| 6 mo | 76 (78.4) | 198 (51.0) | 3.5 (1.8, 6.6) | 0.003 | 134 (70.2) | 454 (59.4) | 1.6 (1.1, 2.3) | 0.01 |
| 9 mo | 66 (68.0) | 208 (53.6) | 1.8 (1.0, 3.3) | 0.04 | 136 (71.2) | 466 (61.0) | 1.6 (1.1, 2.3) | 0.02 |
| 12 mo | 74 (76.3) | 202 (52.1) | 3.0 (1.6, 5.5) | 0.004 | 132 (69.1) | 467 (61.1) | 1.4 (1.0, 2.1) | 0.06 |

^a^ The lowest recorded SBP and DBP were used if there were multiple measurements on the same date

^b^p-values for differences in proportions were calculated from logistic mixed models, with a random intercept for patient. p-values for effect heterogeneity were 0.002, 0.02, 0.61, and 0.03 for the effect of clinic type on controlling high blood pressure at 3, 6, 9, and 12 months, using both in-office and RPM values. The corresponding p-values for in-office measurements were 0.28, 0.50, 0.86, and 0.48.

**Table S3. Systolic Blood Pressure (BP) assessed over 12 Months in patients prescribed remote patient monitoring (RPM) compared with matched controls, stratified by whether clinic used care coordination.**

|  | **Systolic Blood Pressure, mm Hg^a^**  **Care Coordination** | | | | **Systolic Blood Pressure, mm Hg^a^**  **No Care Coordination** | | | | |  |
| --- | --- | --- | --- | --- | --- | --- | --- | --- | --- | --- |
|  | RPM Prescribed N = 97 | Matched controls N = 388 | Difference  (95% CI) | p^b^ | | RPM Prescribed  N = 191 | Matched controls N = 764 | Difference  (95% CI) | p^b^ | |
| ***Baseline: Blood Pressure Metrics (In-Office)*** | | | | |  | | | | |  |
| Index | 142.6 (17.3) | 142.1 (17.6) | 0.4 (-6.6, 7.5) | 0.87 | | 142.8 (20.2) | 140.8 (19.1) | 2.2 (-1.2, 5.6) | 0.20 | |
| ***Outcomes: Blood Pressure Metrics Over Time (In-Office and RPM)^c^*** | | | | |  | | | | |  |
| 3 mo | 131.6 (17.3) | 140.5 (18.6) | -9.0 (14.0, -4.0) | 0.004 | | 135.5 (18.1) | 139.7 (19.0) | -4.0 (-7.2, -0.8) | 0.02 | |
| 6 mo | 132.0 (18.2) | 138.1 (18.7) | -6.2 (-11.3, -1.1) | 0.02 | | 132.9 (18.0) | 137.2 (18.5) | -4.0 (-7.1, -0.9) | 0.01 | |
| 9 mo | 134.7 (20.0) | 136.5 (18.0) | -1.8 (-6.9, 3.3) | 0.43 | | 132.4 (17.7) | 136.2 (18.0) | -3.5 (-6.5, -0.5) | 0.02 | |
| 12 mo | 132.9 (17.3) | 137.3 (17.6) | -4.3 (-9.1, 0.5) | 0.07 | | 132.0 (18.3) | 136.2 (18.8) | -4.0 (-7.1, -0.8) | 0.02 | |

^a^The average daily BP was used if there were multiple measurements on the same date.

^b^p-values for differences in means were calculated from linear mixed models, with a random intercept for patient. p-values for effect heterogeneity were 0.06, 0.41, 0.51, and 0.89 for the effect of clinic type on systolic blood pressure at 3, 6, 9, and 12 months, using both in-office and RPM values. The corresponding p-values for in-office measurements were 0.80, 0.69, 0.52, and 0.03.

^c^Attrition in Care Coordination: 2 (2.1%), 3 (3.1%), 3 (3.1%), and 4 (4.1%) of RPM-prescribed patients and 47 (12.1%), 50 (12.9%), 58 (14.9%), and 50 (12.9%) of matched controls for the systolic blood pressure outcomes assessed at 3, 6, 9, and 12 months, respectively.
Attrition in No Care Coordination: 9 (4.7%), 5 (2.6%), 5 (2.6%), and 6 (3.1%) of RPM-prescribed patients and 38 (5.0%), 33 (4.3%), 33 (4.3%), and 35 (4.6%) of matched controls for the systolic blood pressure outcomes assessed at 3, 6, 9, and 12 months, respectively.

**Table S4. Comparison of RPM SBP measurements with in-office SBP measurements – an investigation for white coat hypertension in patients prescribed RPM.**

| **Time since RPM**  **Prescription** | **n^a^** | **SBP – In-Office^b^**  **Mean (SD)** | **SBP – RPM^c^**  **Mean (SD)** | **Mean Difference**  **(95% CI)** | **p-value^d^** |
| --- | --- | --- | --- | --- | --- |
| Index | 146 | 139.6 (16.6) | 142.6 (19.1) | 3.1 (-0.1, 6.2) | 0.06 |
| 3 mo | 84 | 133.0 (14.0) | 132.2 (15.0) | -0.8 (-4.2, 2.7) | 0.65 |
| 6 mo | 71 | 131.6 (14.1) | 132.1 (13.2) | 0.5 (-3.1, 4.1) | 0.78 |
| 9 mo | 52 | 130.3 (12.5) | 131.7 (19.1) | 1.4 (-4.1, 6.9) | 0.62 |
| 12 mo | 75 | 130.2 (12.4) | 132.6 (15.3) | 2.4 (-1.9, 6.7) | 0.26 |

Abbreviations: RPM – remote physiologic monitoring; SBP – systolic blood pressure; CI – confidence interval

^a^n is a count of patients who had 1) at least one in-office, primary care SBP measured up to 30 days before or after the time point (pegged to each patient’s initial RPM prescription), and (2) at least one RPM SBP measured up to 14 days before or after each patient’s corresponding office visit date.

^b^In-office SBP was calculated using the in-office primary care BP readings from the date closest to the target within 30 days before or after the target date. If multiple SBP measurements were available on that date, their mean was used.

^c^RPM SBP was calculated as the average of all SBP remote readings taken up to 14 days before or after each patient’s corresponding office visit date at each time.

^d^A paired t-test was used to calculate mean differences, 95% CIs, and p-values for the difference between home and in-office SBPs at each time.

**Table S5**. **Time Since Most Recent Blood Pressure Reading Used in 12-Month Outcome Assessments**

|  | **Time Since Most Recent Blood Pressure Measurement, days** | | | |
| --- | --- | --- | --- | --- |
|  | RPM Prescribed N = 288  Median (IQR) | Matched controls N = 1152  Median (IQR) | Difference  (95% CI) | p^a^ |
|  | | | | |
| ***Outcomes: In-Office and RPM*** | 8 (0, 109) | 134 (56, 239) | -126 (-138.4, -113.6) | <0.0001 |
|  | | | | |
| ***Outcomes: In-Office*** | 126 (48, 212) | 134 (56, 239) | -7 (-29.2, 15.2) | 0.54 |

Abbreviations. RPM: remote physiological monitoring; IQR: inner quartile range; CI: confidence interval

^a^p-values were calculated from quantile regression models comparing the median time since most recent blood pressure reading between RPM-prescribed patients and matched controls.
